# The distinct musculoskeletal phenotypes of Type 1 and Type 2 diabetes: a sonographic characterization of enthesopathy burden

**DOI:** 10.1186/s42358-026-00544-0

**Published:** 2026-05-19

**Authors:** Adel Ibrahim Azzam, Taha Ibrahim Aladrosy, Saad Ghanem, Ashraf Abdelsalam Moustafa, Hatem Saadeldin, Yazeed Mohammed AlZahrani, Hilal Rajeh AlZahrani, Afrah Abdulraheem Alshahrani, Abdulaziz Altowairqi, Abdullah Almalki

**Affiliations:** 1 Rheumatology and Rehabilitation Department, Faculty of Medicine, Al-Azhar University, Cairo, Egypt; 2 Rheumatology and Rehabilitation Department, Faculty of Medicine, Al-Azhar University, Damietta, Egypt; 3 Rehabilitation Medicine Department, Armed Forces Center for Health Rehabilitation, Taif, Saudi Arabia

**Keywords:** Diabetes phenotypes, Enthesopathy, Musculoskeletal ultrasound, GUESS score, Metabolic syndrome

## Abstract

**Objective:**

Musculoskeletal complications represent a significant burden in diabetes, yet the distinct presentation between Type 1 (T1DM) and Type 2 Diabetes Mellitus (T2DM) remains under-characterized. This study aimed to evaluate and quantify the burden of rheumatic manifestations and subclinical enthesopathy in these two distinct clinical phenotypes.

**Methods:**

We conducted a cross-sectional analysis of 65 patients (21 T1DM, 44 T2DM). A comprehensive rheumatological evaluation assessed adhesive capsulitis (defined as painful restriction of active and passive motion in ≥ 2 planes), knee osteoarthritis (per American College of Rheumatology clinical classification criteria), cheiroarthropathy, and soft tissue rheumatism. High-resolution musculoskeletal ultrasound evaluated five bilateral entheseal sites using the Glasgow Ultrasound Enthesitis Scoring System (GUESS). Effect sizes (Cohen’s d) and 95% Confidence Intervals (CI) were calculated to assess the magnitude of intergroup differences.

**Results:**

The T2DM phenotype was characterized by older age and higher BMI compared to T1DM. T2DM patients exhibited a markedly higher prevalence of adhesive capsulitis, knee osteoarthritis, and anserine bursitis. Ultrasound revealed a substantially higher enthesopathy burden in the T2DM group, with a mean GUESS score of 6.8 ± 2.1 compared to 1.1 ± 0.2 in T1DM. The effect size for the difference in enthesopathy scores (Cohen’s d = 3.29) exceeded the effect size for the age difference (d = 2.79), suggesting that the observed structural burden may not be fully explained by age alone. Available HbA1c data showed similarly suboptimal glycemic control in both groups.

**Conclusion:**

T2DM was associated with a higher burden of structural entheseal abnormalities and higher GUESS scores compared with T1DM. These findings may reflect the combined influence of obesity, metabolic factors, and age. Interpretation should consider baseline demographic differences between groups and the broad clinical definitions employed.

## Introduction

Diabetes mellitus constitutes a systemic metabolic disease affecting connective tissues, manifesting as diabetic rheumatopathy—a spectrum encompassing limited joint mobility (cheiroarthropathy), adhesive capsulitis, stenosing tenosynovitis, and entheseal pathology [1, 2]. These complications substantially impair quality of life and functional capacity [3, 4].

Pathophysiological mechanisms differ markedly between diabetes types. T1DM pathology centers on non-enzymatic protein glycation [2]. Persistent hyperglycemia promotes advanced glycation end-product (AGE) accumulation in collagen and tendons, inducing cross-linking, tissue stiffness, and impaired remodeling [2, 5].

T2DM is often described as a distinct metabolic-mechanical phenotype. Beyond glycation, patients exhibit insulin resistance, hyperinsulinemia, and obesity [6]. Adipose tissue functions as an endocrine organ, secreting pro-inflammatory cytokines—interleukin-6 and tumor necrosis factor-α—that potentiate tendinopathy and entheseal injury [5, 6]. Hyperinsulinemia may mimic insulin-like growth factor-1 (IGF-1), potentially stimulating osteoblast activity and enthesophyte development at tendon insertions [7–9].

Clinical examination alone inadequately detects early or subclinical enthesopathy [9]. Musculoskeletal ultrasound has emerged as the preferred modality for enthesis visualization [9, 10]. GUESS provides validated semi-quantitative assessment of entheseal structural abnormalities [4, 9, 10].

Despite these recognized differences, few studies have utilized musculoskeletal ultrasound to quantify the specific structural burden in these distinct clinical populations [11, 12]. This investigation aims to characterize and quantify the musculoskeletal phenotypes of T1DM and T2DM, utilizing the Glasgow Ultrasound Enthesitis Scoring System (GUESS) to assess the magnitude of enthesopathy in real-world clinical practice.

## Methods

### Study design and population

This cross-sectional study enrolled 65 confirmed diabetes mellitus patients at a tertiary care center: 21 with T1DM (Group I) and 44 with T2DM (Group II). Inclusion criteria required age ≥ 18 years, diabetes duration ≥ 1 year, and informed consent capability. Exclusion criteria comprised concurrent inflammatory rheumatic diseases (rheumatoid arthritis, spondyloarthritis, psoriatic arthritis), active infections, prior major lower extremity surgery, severe peripheral arterial disease, advanced chronic kidney disease (stages 4–5), active malignancy, and ongoing systemic corticosteroid therapy. The institutional ethics committee approved the protocol; all participants provided written informed consent per Declaration of Helsinki principles.

### Clinical assessment

Participants underwent structured history and comprehensive rheumatological examination. We documented demographics (age, sex, diabetes duration, treatment), anthropometrics (weight, height, BMI), and chronic diabetic complications.

#### Hand assessment

Limited joint mobility was evaluated via prayer sign—inability to approximate palms with extended fingers [1, 2]. Dupuytren’s contracture was assessed by palpating palmar fibrotic bands. Trigger finger was identified by palpable nodules and flexion-extension locking [12, 13].

#### Shoulder assessment

Active and passive range of motion were evaluated for adhesive capsulitis. Adhesive capsulitis was defined clinically as the presence of shoulder pain accompanied by restriction of both active and passive range of motion in at least two planes (typically external rotation and abduction), in the absence of a primary radiographic abnormality of the glenohumeral joint [3, 14]. This clinical definition, while widely used in epidemiological studies of diabetes-associated shoulder pathology [3, 13, 14], is intentionally broad and does not incorporate imaging confirmation or a requirement for a specific threshold of motion loss.

#### Soft tissue rheumatism

Palpation at standardized anatomical landmarks was used to diagnose anserine bursitis (proximal medial tibia), plantar fasciitis (medial calcaneal tubercle), and Achilles tendinitis [1, 5].

#### Neurological screening

Peripheral neuropathy was assessed via sensory testing (10-gram monofilament) and reflex evaluation [12, 15].

#### **Knee assessment**

Knee osteoarthritis was diagnosed clinically using the American College of Rheumatology (ACR) clinical classification criteria for osteoarthritis of the knee [16]. These criteria require the presence of knee pain plus at least three of the following six features: age > 50 years, morning stiffness lasting < 30 min, crepitus on active motion, bony tenderness, bony enlargement, and no palpable warmth of the synovium. This clinical classification approach was selected because radiographic grading was not systematically performed in the study protocol.

Laboratory investigations included erythrocyte sedimentation rate, hemoglobin, white blood cell count, platelet count, and serum uric acid. Glycated hemoglobin (HbA1c) values were extracted from medical records when available.

### Ultrasonographic assessment and GUESS scoring

A single experienced sonographer, blinded to diabetes type, performed high-resolution musculoskeletal ultrasound using a high-frequency linear transducer (6–18 MHz). Ultrasound acquisition settings (gain, depth, and focus) were standardized as much as possible across all examinations to ensure comparability. Five bilateral entheseal sites were evaluated per GUESS methodology [4, 9, 10]: quadriceps tendon (superior patellar pole), proximal patellar ligament (inferior patellar pole), distal patellar ligament (tibial tuberosity), Achilles tendon (superior calcaneal pole), and plantar fascia (inferior calcaneal pole). Each site received scores for tendon thickening, bursitis, enthesophytes, and cortical erosions (1 point each) [4, 9, 10]. Cortical erosions were defined as a cortical break at the enthesis and were only recorded when clearly visible in two perpendicular planes (longitudinal and transverse), in accordance with standard ultrasound definitions. Total GUESS scores (range 0–36) quantified structural entheseal pathology.

**Fig. 1:**
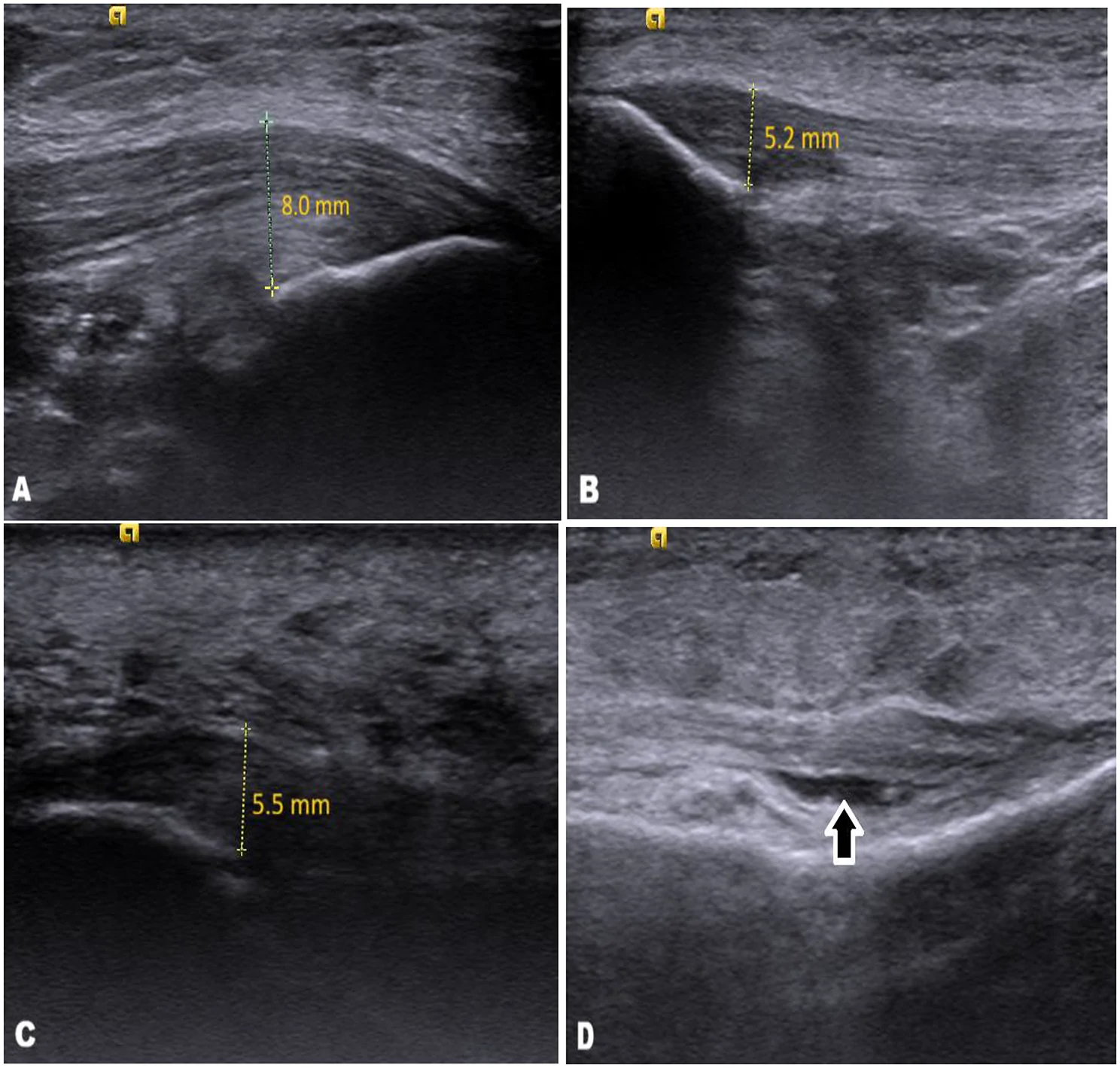
Representative gray-scale ultrasound images demonstrating structural entheseal abnormalities in patients with type 2 diabetes mellitus. (**A**) Quadriceps tendon showing significant thickening (8.0 mm) at the superior patellar insertion. (**B**) Proximal patellar tendon thickening (5.2 mm) at the inferior patellar pole. (**C**) Plantar fascia thickening (5.5 mm) at the calcaneal origin. (**D**) Longitudinal scan revealing anechoic fluid collection (arrow) consistent with pes anserine bursitis. Note: Thickness measurements were obtained strictly at the entheseal insertion sites in accordance with the GUESS protocol

Figure 1 illustrates representative sonographic examples of enthesopathology observed in our cohort of patients with type 2 diabetes.

### Statistical analysis

Data analysis was performed using SPSS version 25.0 (IBM Corp., Armonk, NY, USA). Continuous variables are expressed as mean ± standard deviation (SD), and categorical variables as frequencies and percentages. Group comparisons were conducted using independent-samples t-tests for continuous variables and Chi-square tests for categorical variables. To quantify the magnitude of between-group differences beyond statistical significance, mean differences with 95% confidence intervals (CI) and effect sizes (Cohen’s d) were calculated. Effect sizes were interpreted as small (0.2), medium (0.5), and large (> 0.8).

Given the relatively small sample size, particularly in the T1DM group, the study may have been underpowered to detect small to moderate differences in certain clinical variables. Accordingly, non-significant findings should be interpreted with caution and not considered evidence of equivalence between groups. Owing to sample size constraints, multivariable regression analysis was not performed.

## Results

### Demographics

The groups exhibited significant demographic differences (Table 1). T2DM patients were considerably older (mean 50.1 ± 8.44 vs. 24.5 ± 10.56 years, *p* < 0.001) with later diabetes onset (41.4 ± 10.07 vs. 17.3 ± 7.17 years, *p* < 0.001). Obesity characterized the T2DM cohort (mean BMI 32.7 ± 6.0 vs. 27.5 ± 6.3 kg/m², *p* = 0.01).

### Glycemic control

HbA1c data were available for a subset of participants (15 of 21 T1DM patients [71.4%] and 30 of 44 T2DM patients [68.2%]). In this subset, mean HbA1c was 8.9 ± 1.6% in the T1DM group and 8.4 ± 1.8% in the T2DM group (mean difference 0.5%, 95% CI − 0.6 to 1.6; Cohen’s d = 0.29; *p* = 0.38). These values indicate suboptimal glycemic control in both groups, with no statistically significant difference between them. However, given the incomplete data availability, these results should be interpreted with caution and cannot be used to draw definitive conclusions about the relationship between glycemic control and musculoskeletal outcomes in this cohort.

### Clinical musculoskeletal manifestations

T2DM patients demonstrated significantly higher musculoskeletal complication prevalence across most categories. Adhesive capsulitis affected 81.8% of T2DM versus 57.1% of T1DM patients (*p* = 0.038). Knee osteoarthritis showed greater disparity (84% vs. 47.6%, *p* = 0.002), likely reflecting higher T2DM BMI.

Anserine bursitis exhibited the most striking difference (86.4% vs. 14.2%, *p* < 0.001), probably reflecting obesity-associated mechanical stress. Plantar fasciitis occurred in 52.2% of T2DM versus 14.2% of T1DM patients (*p* = 0.003). Achilles tendinitis affected 22.7% of T2DM patients but no T1DM patients (*p* = 0.022).

Cheiroarthropathy prevalence differed significantly (38.6% vs. 9.5%, *p* = 0.015), potentially reflecting prolonged hyperglycemia effects on collagen. Hand complications—carpal tunnel syndrome (27.2% vs. 14.2%, *p* = 0.245) and trigger finger (25.0% vs. 14.2%, *p* = 0.335)—showed higher T2DM prevalence without statistical significance.

Peripheral neuropathy demonstrated similar prevalence (76.1% T1DM vs. 75% T2DM, *p* = 0.921), suggesting common glycemic origin independent of diabetes type. 

### Ultrasonographic findings and GUESS scores

Ultrasound revealed a substantial burden of enthesopathy in the T2DM phenotype (Table 2). The total GUESS score was 6.8 ± 2.1 in T2DM compared to 1.1 ± 0.2 in T1DM. The effect size for this difference was very large (d = 3.29), indicating a markedly greater structural burden in the T2DM group. Retrocalcaneal bursitis was a predominant feature in T2DM (36.4%), whereas it was rare in T1DM (4.8%).

Site-specific findings demonstrated significant intergroup differences. Retrocalcaneal bursitis predominated in T2DM (36.4% vs. 4.8%, p < 0.001). Infrapatellar bursitis affected 29.5% of T2DM versus 4.8% of T1DM patients (p = 0.023). Quadriceps enthesophytes showed highest prevalence (72.7% T2DM vs. 33.3% T1DM, p = 0.003).

**Table 1:** Demographic and clinical characteristics

Variable	Type 1 DM (*n* = 21)	Type 2 DM (*n* = 44)	Mean Difference (95% CI)	Effect Size (Cohen’s d)	*p* value
Demographics					
Age (years), mean ± SD	24.5 ± 10.56	50.1 ± 8.44	25.6 (20.5 to 30.8)	2.79	0.001
BMI (kg/m²), mean ± SD	27.5 ± 6.3	32.7 ± 6.0	5.2 (2.0 to 8.4)	0.85	0.010
HbA1c (%), mean ± SD*	8.9 ± 1.6 (*n* = 15)	8.4 ± 1.8 (*n* = 30)	0.5 (− 0.6 to 1.6)	0.29	0.38
Male, n (%)	14 (66.9%)	7 (15.9%)	—	—	0.001
Female, n (%)	7 (33.1%)	37 (84.1%)	—	—	0.001
Clinical Findings					
Adhesive Capsulitis, n (%)	12 (57.1%)	36 (81.8%)	—	—	0.038
Knee osteoarthritis, n (%)	10 (47.6%)	37 (84.0%)	—	—	0.002
Anserine bursitis, n (%)	3 (14.2%)	38 (86.4%)	—	—	< 0.001
Plantar fasciitis, n (%)	3 (14.2%)	23 (52.2%)	—	—	0.003
Achilles tendinitis, n (%)	0 (0%)	10 (22.7%)	—	—	0.022
Cheiroarthropathy, n (%)	2 (9.5%)	17 (38.6%)	—	—	0.015
Carpal tunnel syndrome, n (%)	3 (14.2%)	12 (27.2%)	—	—	0.245
Trigger finger, n (%)	3 (14.2%)	11 (25.0%)	—	—	0.335
Peripheral neuropathy, n (%)	16 (76.1%)	33 (75.0%)	—	—	0.921

Abbreviations: BMI = body mass index; CI = confidence interval; SD = standard deviation. Effect sizes interpreted as: 0.2 small, 0.5 medium, > 0.8 large

*HbA1c data were available for 15/21 (71.4%) T1DM and 30/44 (68.2%) T2DM patients

**Table 2:** Ultrasonographic entheseal involvement according to GUESS criteria

Ultrasound Variable	Type 1 DM (*n* = 21)	Type 2 DM (*n* = 44)	Mean Difference (95% CI)	Effect Size (Cohen’s d)	*p* value
Total GUESS score, mean ± SD	1.1 ± 0.2	6.8 ± 2.1	5.7 (5.1 to 6.3)	3.29	0.001
Retrocalcaneal bursitis, n (%)	1 (4.8%)	16 (36.4%)	—	—	0.001
Infrapatellar bursitis, n (%)	1 (4.8%)	13 (29.5%)	—	—	0.023
Quadriceps enthesophyte, n (%)	7 (33.3%)	32 (72.7%)	—	—	0.003
Achilles enthesophyte, n (%)	2 (9.5%)	28 (63.6%)	—	—	0.001
Plantar fascia thickening, n (%)	3 (14.3%)	31 (70.5%)	—	—	0.001

Abbreviations: CI = confidence interval; GUESS = Glasgow Ultrasound Enthesitis Scoring System; SD = standard deviation. Bold indicates the primary outcome measure showing an effect size exceeding that of demographic confounders

## Discussion 

This study characterizes the distinct musculoskeletal phenotypes of T1DM and T2DM. Our findings suggest that T2DM is associated with a ‘metabolic-mechanical’ phenotype characterized by a higher burden of structural enthesopathy compared with T1DM

The most critical finding is the magnitude of the entheseal burden. While the T2DM cohort was significantly older (Effect Size *d* = 2.79), the difference in GUESS scores was even more profound (Effect Size *d* = 3.29). This suggests that the observed increase in enthesopathy scores in T2DM may not be fully explained by aging alone and could reflect the combined influence of obesity, metabolic factors, and mechanical load; however, residual confounding cannot be excluded

The marked elevation in GUESS scores in T2DM is consistent with a substantially greater structural enthesopathy burden in this phenotype [4]. Hyperinsulinemia common in T2DM may critically contribute to this phenotype [6, 7]. Given insulin’s structural similarity to IGF-1, elevated circulating insulin may enhance osteoblast activity, explaining markedly higher enthesophyte prevalence in T2DM compared to insulin-deficient T1DM [7]. These findings may indicate a predominantly structural/proliferative pattern; however, inflammatory activity cannot be excluded in the absence of power Doppler assessment [7–10]

The exceptionally high anserine bursitis (86.4%) and knee osteoarthritis (84%) rates in T2DM highlight mechanical loading impact. With mean BMI 32.7 kg/m² indicating clinical obesity, these patients experience substantial additional lower extremity compressive forces [5]. The pes anserinus bursa, cushioning hamstring tendon insertions, undergoes chronic compression and friction in obese patients, predisposing to bursitis [6]. Conversely, the T1DM group (mean BMI 27.5 kg/m²) exhibited minimal bursitis (14.2%) despite longer disease duration from early adolescence

This suggests that while glycation and AGE accumulation contribute to tendon stiffness in both groups, weight-related mechanical stress primarily drives bursitis and acute soft tissue inflammation [5, 6]

Peripheral neuropathy prevalence was remarkably similar between groups (76.1% T1DM vs. 75% T2DM, *p* = 0.921), representing the sole musculoskeletal variable without significant intergroup difference. This similarity may reflect shared mechanisms related to chronic hyperglycemia and microvascular injury across both diabetes phenotypes [17]. The available HbA1c data, though incomplete, support this interpretation by demonstrating similarly suboptimal glycemic control in both groups (8.9% vs. 8.4%, *p* = 0.38). This comorbidity exacerbates joint and tendon damage by impairing proprioception and diminishing protective pain sensation, creating ongoing tissue damage and functional decline [1, 17]

An important methodological consideration is the potential for imperfect blinding of the ultrasound examiner. Although the sonographer was formally blinded to diabetes type, the distinct clinical phenotypes of T1DM and T2DM patients—with the T1DM group being substantially younger (mean age 24.5 vs. 50.1 years) and leaner (mean BMI 27.5 vs. 32.7 kg/m²)—may have allowed the examiner to infer the diabetes type during the examination. This unintentional unblinding could theoretically introduce observer bias in the scoring of subjective GUESS components such as tendon thickening or enthesophyte grading. However, several factors mitigate this concern: the GUESS scoring system relies on predefined structural criteria with objective measurement thresholds (e.g., tendon thickness in millimeters), ultrasound acquisition settings were standardized, and cortical erosions were only recorded when confirmed in two perpendicular planes. Nevertheless, this potential source of bias should be acknowledged, and future studies should consider implementing full blinding protocols—such as obscuring the patient’s body habitus from the sonographer’s view or employing independent image readers—to minimize this risk

These findings suggest that musculoskeletal ultrasound may have a potential role in the assessment of structural entheseal involvement in patients with T2DM. Significantly elevated T2DM GUESS scores indicate prevalent subclinical tendon and entheseal injuries [4]. Early detection of these structural abnormalities through GUESS-based ultrasound could facilitate preventive interventions—weight management, targeted physical therapy, orthotic support, and enhanced glycemic control—to arrest progression and prevent severe complications including tendon ruptures and debilitating adhesive capsulitis [1, 4, 9, 10]

Healthcare providers should maintain high vigilance for musculoskeletal complications in diabetic patients, especially those with T2DM, obesity, and prolonged disease duration [4, 12]. Systematic assessment for common musculoskeletal complications may be considered as part of comprehensive diabetes care, particularly in patients with T2DM and obesity

Several limitations of this study must be acknowledged. First, the significant differences in age and BMI between the T1DM and T2DM groups reflect the natural epidemiology of these phenotypes but may act as confounding factors for entheseal pathology. While effect size calculations suggest that the magnitude of the GUESS score difference may exceed what would be expected from age alone, this does not replace multivariable adjustment, and residual confounding cannot be excluded. Accordingly, the present findings should be interpreted as associative observations rather than evidence of causal inference. Second, the definition of adhesive capsulitis used in this study was based on clinical examination (painful restriction of active and passive motion in ≥ 2 planes) without imaging confirmation or a specified threshold of range-of-motion loss. This broad operational definition may have overestimated the true prevalence of adhesive capsulitis in both groups, particularly given that early or mild shoulder stiffness—which may not represent true capsular pathology—could have been captured. The high prevalence observed (57.1% in T1DM and 81.8% in T2DM) should therefore be interpreted in light of this broad case definition, and future studies should consider incorporating standardized goniometric thresholds or imaging criteria to improve diagnostic specificity. Third, HbA1c data were available for only a subset of participants (71.4% of T1DM and 68.2% of T2DM patients). While the available data suggest similarly suboptimal glycemic control in both groups (mean HbA1c 8.9% vs. 8.4%, *p* = 0.38), the incomplete ascertainment prevents definitive correlation analysis between long-term hyperglycemia and GUESS scores. Selection bias in HbA1c availability cannot be excluded, and the subset analysis may not be representative of the entire cohort. Fourth, the GUESS score primarily assesses structural damage; the exclusion of Power Doppler evaluation means that active subclinical inflammation may have been underestimated. Finally, formal intra-observer reliability testing was not performed. Despite these limitations, the study highlights a substantial musculoskeletal burden in T2DM

## Conclusion 

Type 2 diabetes mellitus was associated with a higher prevalence of musculoskeletal complications and a greater burden of ultrasonographic structural entheseal abnormalities compared with type 1 diabetes in this cohort. Although these differences may partly reflect variations in age, body mass index, and metabolic profile between groups, the observed magnitude of structural involvement underscores the importance of musculoskeletal assessment in patients with T2DM. Musculoskeletal ultrasound may serve as a valuable tool for detecting subclinical structural abnormalities in this population. Future longitudinal studies incorporating matched cohorts and metabolic biomarkers are warranted to further elucidate the mechanisms and progression of diabetic enthesopathy

## Data Availability

The datasets used and/or analyzed during the current study are available from the corresponding author on reasonable request.
